# Automated and Accurate Detection of Soma Location and Surface Morphology in Large-Scale 3D Neuron Images

**DOI:** 10.1371/journal.pone.0062579

**Published:** 2013-04-24

**Authors:** Cheng Yan, Anan Li, Bin Zhang, Wenxiang Ding, Qingming Luo, Hui Gong

**Affiliations:** 1 Britton Chance Center for Biomedical Photonics, Huazhong University of Science and Technology-Wuhan National Laboratory for Optoelectronics, Wuhan, China; 2 MoE Key Laboratory for Biomedical Photonics, Department of Biomedical Engineering, Huazhong University of Science and Technology, Wuhan, China; Imperial College London, United Kingdom

## Abstract

Automated and accurate localization and morphometry of somas in 3D neuron images is essential for quantitative studies of neural networks in the brain. However, previous methods are limited in obtaining the location and surface morphology of somas with variable size and uneven staining in large-scale 3D neuron images. In this work, we proposed a method for automated soma locating in large-scale 3D neuron images that contain relatively sparse soma distributions. This method involves three steps: (i) deblocking the image with overlap between adjacent sub-stacks; (ii) locating the somas in each small sub-stack using multi-scale morphological close and adaptive thresholds; and (iii) fusion of the repeatedly located somas in all sub-stacks. We also describe a new method for the accurate detection of the surface morphology of somas containing hollowness; this was achieved by improving the classical Rayburst Sampling with a new gradient-based criteria. Three 3D neuron image stacks of different sizes were used to quantitatively validate our methods. For the soma localization algorithm, the average recall and precision were greater than 93% and 96%, respectively. For the soma surface detection algorithm, the overlap of the volumes created by automatic detection of soma surfaces and manually segmenting soma volumes was more than 84% for 89% of all correctly detected somas. Our method for locating somas can reveal the soma distributions in large-scale neural networks more efficiently. The method for soma surface detection will serve as a valuable tool for systematic studies of neuron types based on neuron structure.

## Introduction

The brain is a complex network comprised of hundreds of thousands of inter-connected neurons with varying morphologies. Specific brain functions rely on specific types of neurons. However, the exact numbers and types of neurons in the brain are still a mystery [1], [2]. It is well known that neural structure, including soma location and surface morphology, is one of the most important characteristics for discriminating different types of neurons [2]. Hence, accurate soma localization and surface detection will further exploration of the brain by revealing more neuron types.

The size of the brain of the commonly used model organism, the mouse, is in the centimeter scale, and the soma sizes span a few microns to dozens of microns [3]. To investigate the details of the structures of single neurons in large-scale neural networks or the whole brain, several types of neuron labeling techniques [4], [5] and high-resolution whole brain imaging methods [6], [7] have been proposed recently. Consequently, high resolution and extremely large datasets of neuron structures have been generated that will undoubtedly become a tremendous challenge for traditional analysis methods, such as manual segmentation or semi-automated segmentation with human intervention as these methods are time consuming. As a result, there is an urgent need for the development of automated methods for soma detection that include both soma localization and surface detection [8].

Significant progress on soma detection in 3D neuron images has been achieved using several methods. In phase-contrast microcopy, the somas often have higher contrast than the neurites. In these images, somas can be easily detected by simple threshold methods [9], [10], but extension of this method to images captured by other types of microscopy is difficult. In fluorescence imaging, somas and neurites can often be imaged in separate image channels, and this can simplify the problem into soma detection without neurite intervention. Several methods have been proposed for this type of image [11]–[17]. However, these methods cannot be used for images in which the somas and other structures exist in the same image channel. Several other methods have been employed to solve this problem. For example, in neuron culture images, 2D soma detection can be achieved by combining Gaussian smoothing, morphological top-hat, morphological open, and interactive thresholds [18]. These types of methods have proven difficult to extend to 3D due to their complexity. Some other techniques, such as Laplacian-of-Gaussian filter [19] and mean-shift clustering [20], may be implemented directly in 3D space to detect the candidate soma center by finding the peak response or local maximum intensity. But when some irregular hollowness exists inside somas, these methods may detect more than one soma candidate for each soma (over-segmentation). In 3D confocal or bright-filed images, somas can be detected by 3D morphological closing [21]. However, the computational efficiency is the main obstacle for the application of this method, and this method cannot obtain soma surface information either. To improve the computational efficiency of 3D processing, detection of the soma area in the three 2D orthogonal projection images of the original 3D image stack using 2D morphological closing has been proposed [22]–[24]. Then the 3D soma locations could be obtained by backprojecting three projection images including the soma area into 3D image space. Here, we refer to this type of method as the 2.5 D morphological method. Although this type of method has the advantage of high efficiency and high accuracy, it has, to date, only been used for small image stacks containing only one soma. When multiple somas exist in a large-scale image stack, some somas may appear as cluster areas in projection images, and this is hard to overcome with 2.5 D morphological methods. Furthermore, 2.5 D morphological methods cannot provide any soma surface information. The classical Rayburst Sampling has demonstrated advanced abilities for the surface detection of complex blob-like structures [25]–[27]. Originally, this algorithm was proposed for the accurate surface reconstruction of complex spine structures in fluorescence images; but it was only suitable for the surface detection of solid blob-like objects due to its simple criteria based on the intensity threshold. When some hollowness exists inside the somas, this method will not work.

**Figure 1:**
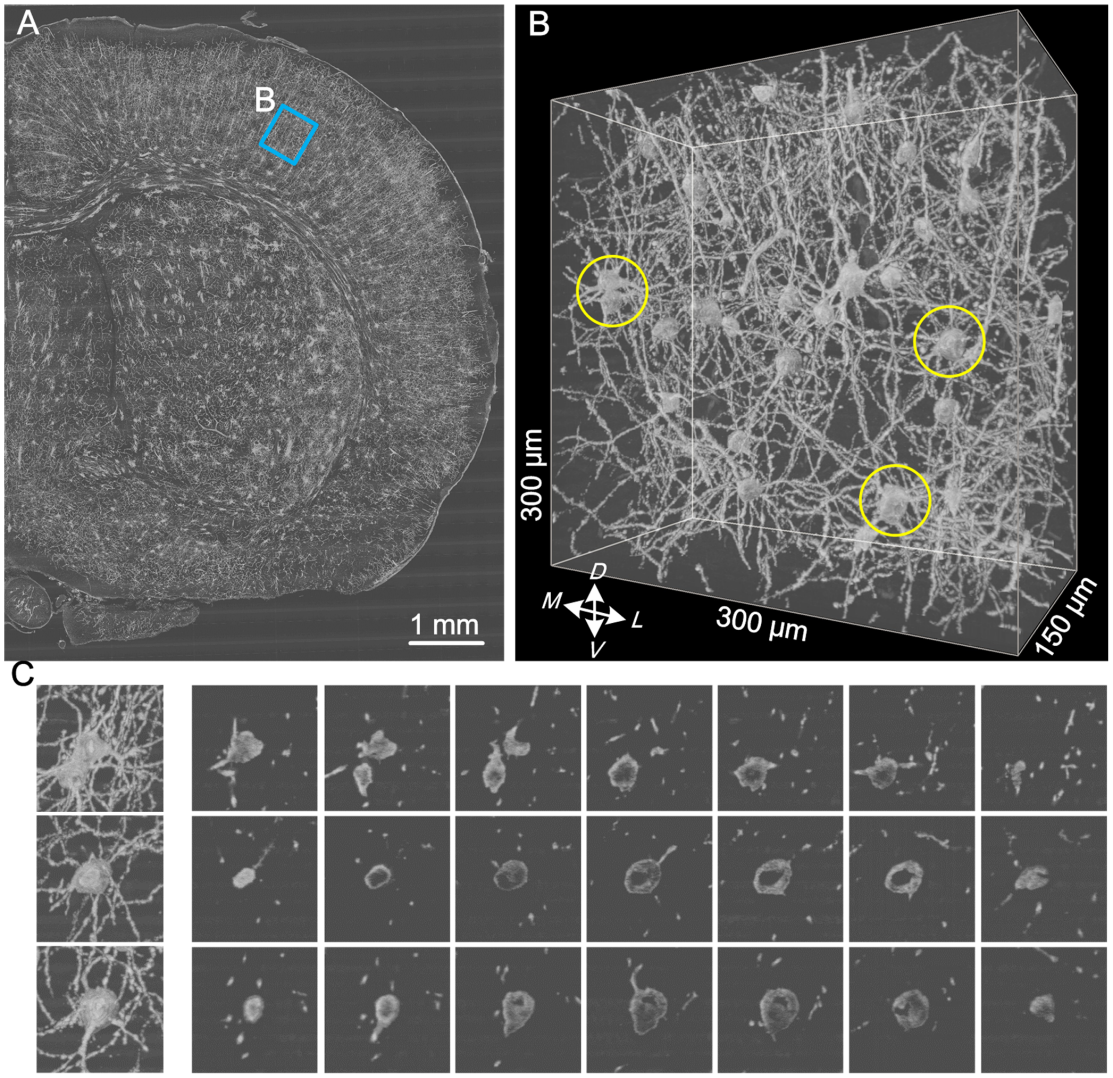
The main features of images acquired from a Golgi-stained whole mouse brain using MOST. (A) A half of a coronal slice used for soma size measurement. (B) The local 3D image stack including random soma distributions and comparatively dense neurites from (A). The three somas surrounded with yellow circles correspond to the three somas in (C). (C) Examples showing the hollowness of somas in (B). The first column shows the projection images of the sub-stacks containing the three somas. The right image sequence shows discontinuous sections with fixed interval (10 µm) in each sub-stack.

Golgi staining, which is a classical, sparsely labeling method for the study of neural structure, can randomly label a few neurons in a whole brain [4]. Recently, many researchers have begun to use this method to acquire neuron image datasets in large-scale brain tissue or even whole mouse brains [6], [7], [28]. Hence, the development of an automated method for soma detection that could be applied to this image dataset will be of great significance. For example, a set of high-resolution neuron image datasets for the whole Golgi-stained mouse brain has been acquired using Micro-Optical Sectioning Tomography (MOST) [6], [29]. Figure 1 demonstrates some typical image features of this dataset. Taken together, three main challenges exist for automated soma location and surface detection in large-scale image stacks. (i) Variable soma sizes. Based on manual size measurements of 72 somas in a large-scale image stack (Fig. 1 A), soma sizes vary from 15 µm to 27 µm, and the average soma size is about 21 µm. (ii) Interference from dense neurites. Although Golgi staining is considered to be a sparse labeling technique, these image datasets are still composed of comparatively dense neurites punctuated by random somas (Fig. 1 B). (iii) Somas containing hollowness. Similar to other traditional tissue staining methods, Golgi staining results in some hollowness (Fig. 1 C) inside most somas due to uneven staining.

Here, we use the dataset above as an example to describe our automated and accurate method for soma detection in large-scale 3D neuron image stacks that contain a relatively sparse distribution of somas. Two main contributions were made by the proposed method.

First, we proposed new method for accurate location of unevenly-stained somas of variable sizes. To locate somas in a relatively small stack, we improved the 2.5 D morphological method [22]–[24] by integrating multi-scale morphological close with an adaptive threshold during the 2D soma detection procedure. For a relatively large stack, both the original 2.5 D morphological method and our improved method could not overcome the soma clusters in projection images. Therefore, assisted by the idea of image deblocking with overlap between adjacent sub-stacks, our improved method was used in each sub-stack to avoid the problem above. Finally, we can locate all somas in the original whole image stack by filtering those repeatedly located somas in all sub-stacks using a fusion operation based on certain criteria.

Second, during the soma location procedure, a volume intersection larger than each soma volume could be obtained by backprojecting three 2D orthogonal projection images into 3D image space. Assisted by the constraint of this volume intersection, accurate surface detection of somas containing hollowness was achieved by integrating new gradient-based criteria with original Rayburst Sampling [25]–[27].

We applied the proposed method to three neuron image stacks of different sizes, and we found that this method was effective in terms of soma localization and surface detection.

## Materials and Methods

### Imaging Data

The image datasets used in present work come from an adult mouse. First, the entire brain was extracted and stained and embedded using the modified Golgi-cox method [4]. Then, the prepared whole brain specimen was imaged with the MOST system [6] to obtain the whole brain dataset with sub-micron voxel resolution (0.35×0.35×1 µm^3^). Basic preprocessing including periodic noise removal and luminance balance was carried out on the acquired images prior to soma detection [6]. In this work, we chose three image stacks of different sizes from the cerebral cortex to test our method. We use the first image stack (Fig. 1 B) to illustrate key steps in our algorithm. In this work, all animal experiments followed the procedures approved by the Institutional Animal Ethics Committee of Huazhong University of Science and Technology.

### Method Overview

**Figure 2:**
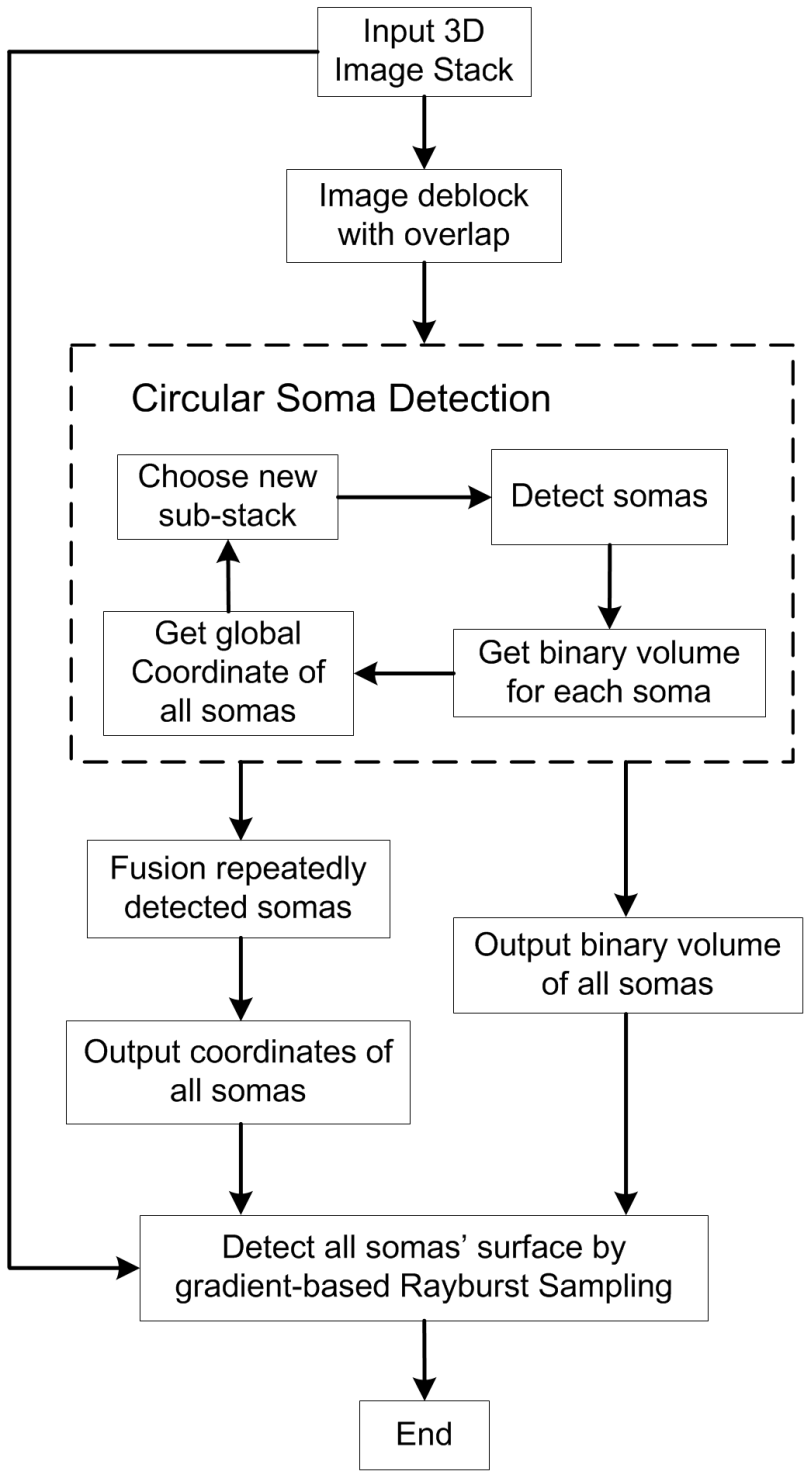
The complete procedure for the proposed method.

The complete procedure for the proposed method is shown in Fig. 2. A large-scale 3D image stack was first deblocked into several sub-stacks with a certain overlap between adjacent sub-stacks. Then, soma localization, computation of soma binary volumes and calculation of potential soma centroids were carried out successively for each sub-stack. After traversing of all sub-stacks was completed, many repeatedly detected somas in adjacent sub-stacks were fused based on certain criteria to obtain the final location of all somas. A binary image stack including the volumes of all somas was also output in this step. Assisted by the original 3D image stack and the binary image that included the volumes of all somas, an improved Rayburst Sampling (gradient-based Rayburst Sampling) was used for the surface detection of all somas.

Notably, intermediate results will be given for most of the procedure steps in the following sections.

### Deblocking with Overlap between Adjacent Sub-stacks

**Figure 3:**
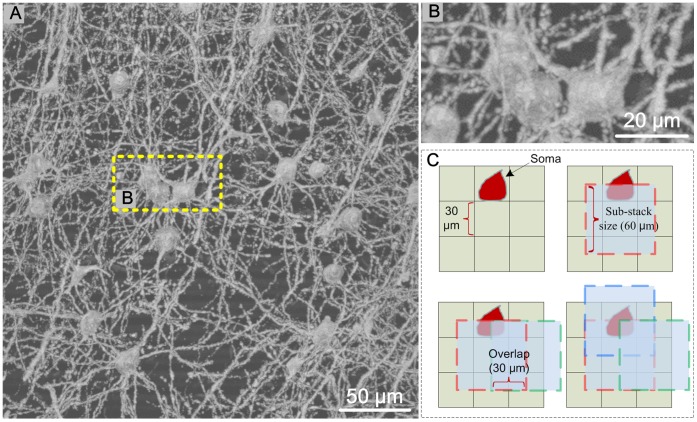
The deblocking strategy used for soma detection in a large-scale image stack. **(A)** The maximum intensity projection of the original image stack (thickness: 150 µm). **(B)** Enlarged view of the dashed box in **A**. (C) Schematic diagram of our deblocking strategy. For simplicity, we only show our deblocking strategy using the 2D version of our method. We drew numerous square grids on a 2D image, and the side length of the square grids was 30 µm. The side length for each sub-stack was 60 µm. The overlap between adjacent sub-stacks was 30 µm. Three adjacent sub-stacks (red, green, and blue dashed boxes) around the red soma were used.

If an image stack contains only one soma, soma detection can be carried out successfully with using the 2.5 D morphological method [22]–[24]. Even if some hollowness exists in the soma due to uneven staining, the 2.5 D morphological method has the potential to solve the problem with some adaptable modifications. However, for large-scale image stacks that include multiple somas, intensity projections along three orthogonal orientations cause many somas to appear as clusters in the projection images (Fig. 3 (A, B)). In this case, the 2.5 D morphological method will not work.

Given that Golgi staining only labels a few neurons in brain tissue, those somas that appear as clusters in projection images may often be separated from each other in 3D space. We deblocked the whole image stack into several sub-stacks (Fig. 3 C). Then, the 2.5 D morphological method was used to find the somas in each sub-stack. Specifically, to avoid missing somas due to the deblocking, we allowed some overlap between adjacent sub-stacks. The overlap size needed to be larger than the average size of the somas (here, we set the overlap to 30 µm). Example results are shown in Fig. 3 C; although the red soma was cut by the red and green sub-stacks (dashed box), it was still embraced by the blue sub-stack. Thus, our deblocking strategy can avoid missing somas.

### Soma Location in Local Sub-stacks

**Figure 4:**
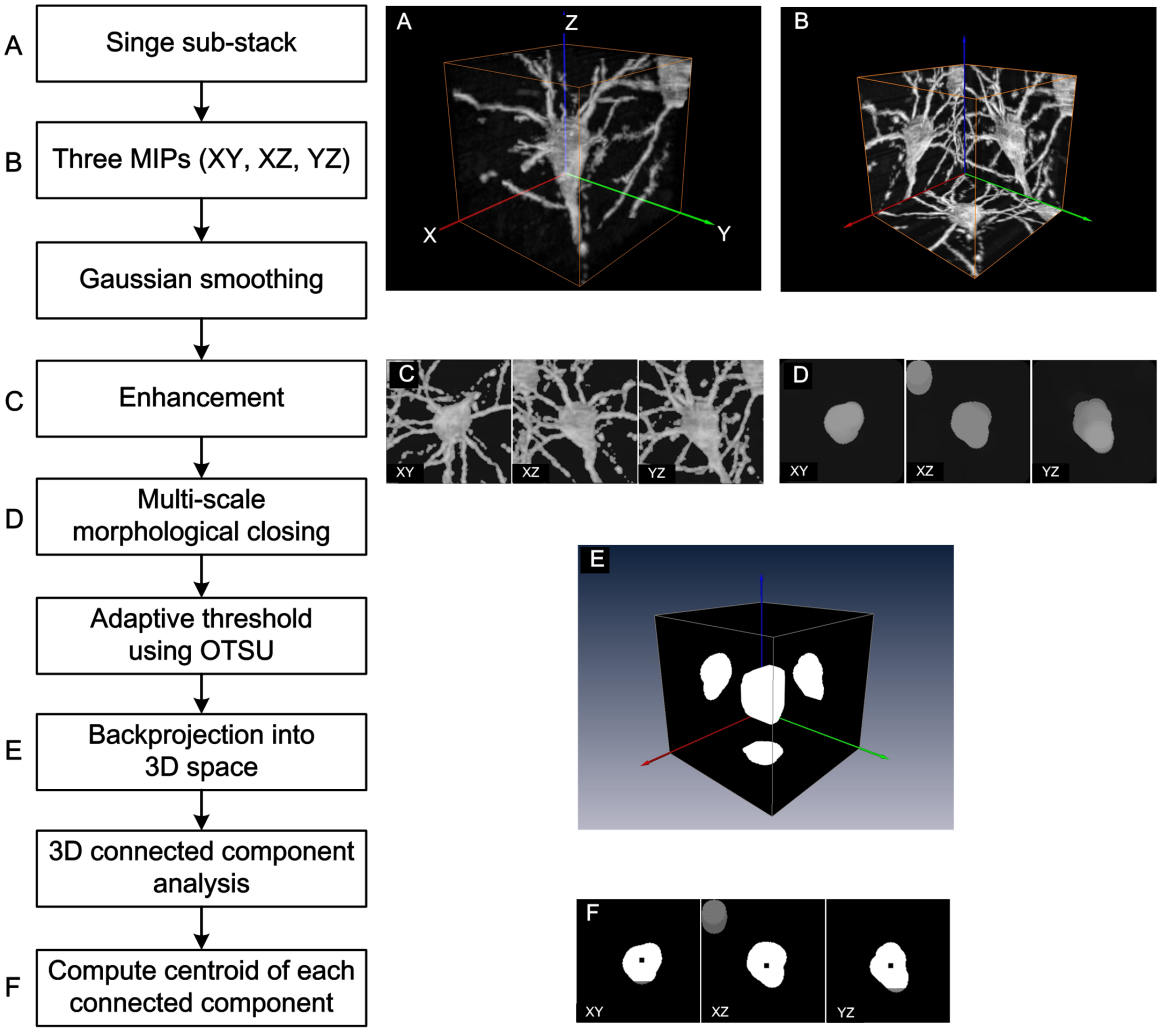
Algorithm for locating a soma in a local sub-stack. (A) The original image sub-stack. (B) The maximum intensity projection image of the sub-stack in (A) along three orthogonal orientations. (C) The images after Gaussian smoothing. (D) The images after grayscale morphological closing. (E) The binary volume intersection including the soma volume achieved by the backprojection of the three projection images into 3D image space. (F) The images including the soma centroid obtained by connected component analysis and computation of the center of mass.

To acquire soma locations in each sub-stack, a strategy similar to the 2.5 D morphological method was adopted. The complete procedure is shown in Fig. 4. First, maximum intensity projections along the three orthogonal directions were conducted to obtain three projection images (I*xy*, I*xz* and I*yz*). Then, Gaussian smoothing, intensity enhancement, grayscale morphological closing and adaptive thresholding were performed successively for each projection image. The three processed projection images were then backprojected into 3D image space to find potential somas using connected component analysis. The proposed method made improvements in two main aspects compared with the 2.5 D morphological method.

1. Gaussian smoothing. The novel staining and embedding method for whole brain tissue might result in uneven staining of somas. In this case, the soma would present with uneven grayscale intensities in the three projections (Fig. 4 B). Gaussian smoothing was introduced to improve the uniformity of soma intensities and was followed by image enhancement (Fig. 4 C).
2. Multi-scale morphological closing. For a large-scale image stacks that included multiple somas, the soma sizes may change in a certain range. Smaller soma may have the similar sizes as parts of the apical dendrites of some pyramidal neurons. Thus, it was difficult to detect all somas using a uniform morphological template size. Smaller template sizes may produce many false positive somas, and larger template sizes would miss some small somas. Thus, a multi-scale morphological closing was used to detect somas of varying sizes in the present work. The detailed steps for our multi-scale morphological closing are as follows.

Based on the statistical results from measuring the sizes of a large number of somas, we set the sizes of the circular template used in the morphological closing as the range of 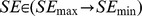. 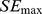 is between the minimum and maximum soma size. 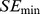 is slightly smaller than the minimum soma size. The template size decreased by a defined step to implement the multi-scale morphological closing mentioned above. In the present work, 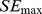 and 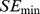 were set to 21 µm and 12 µm, respectively, and the step size for decreases in template sizes was set to 2 µm.

For each circular template size, morphological closing was carried out on three projection images (Fig. 4 D) followed by adaptive threshold using the OTSU method (Fig. 4 E) [30].

When somas were in the current sub-stack, a series of volume intersections 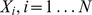 (Fig. 4 E) were produced after the three projection images were backprojected into 3D image space. Assisted by connected component analysis, we obtained a series of soma centroids 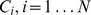 (Fig. 4 F) by averaging the coordinates of all voxels in each component. Once any soma was located in current sub-stack, the detection procedure was terminated. Otherwise, the procedure was repeated after decreasing the template size by the defined step.

In present work, larger somas could often be detected using the max template size, while smaller ones could be detected after 2–3 times of reductions in template size.

### Fusion of the Repeatedly Detected Somas

**Figure 5:**
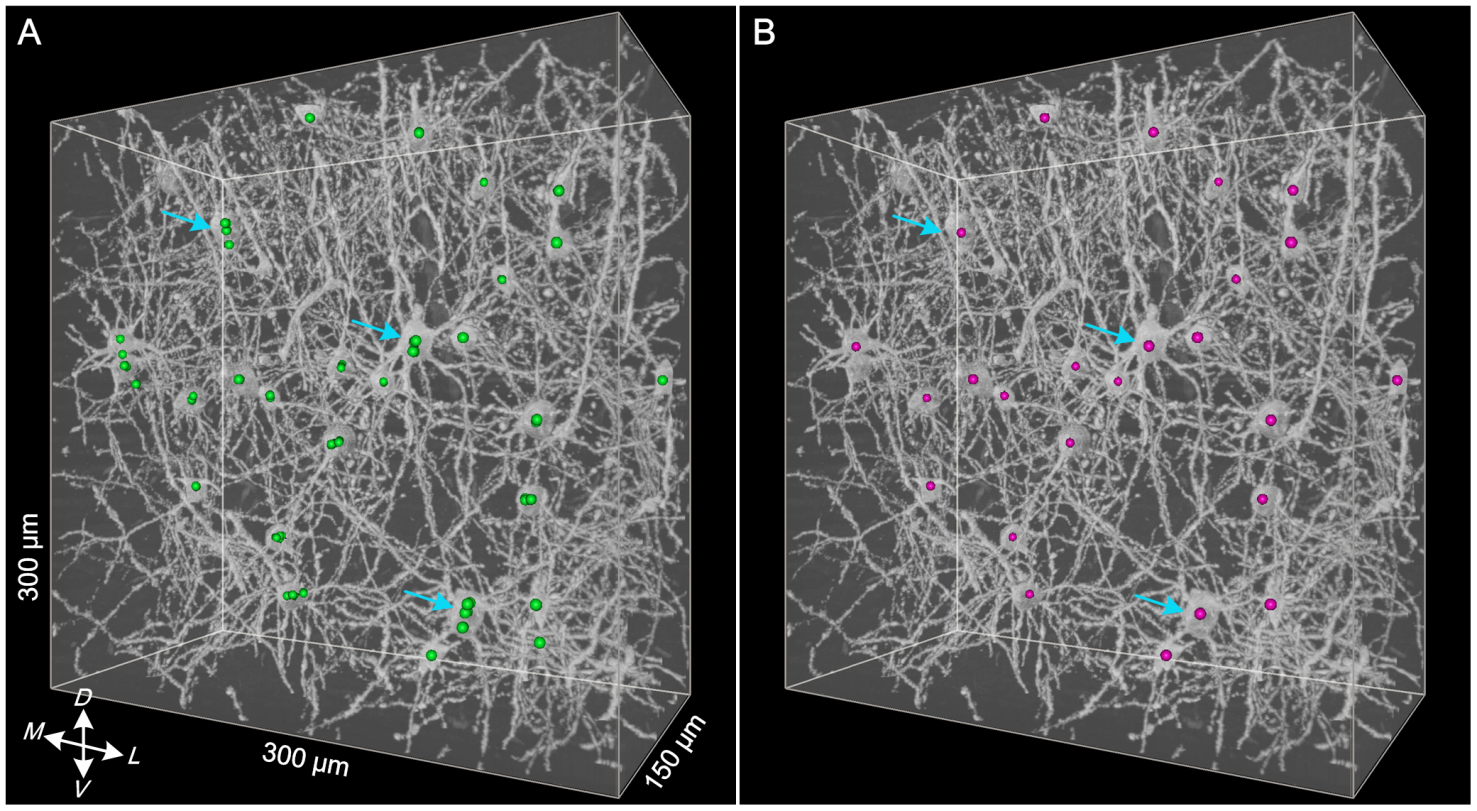
Fusion of the repeatedly detected somas. (A) The candidate somas (green spheres) overlaid on the original image stack before soma fusion. (B) The final somas (red spheres) overlaid on the original image stack after soma fusion. The blue arrows show some typical somas before and after fusion.

Due to the deblocking strategy used in our method, some somas might exist in many adjacent sub-stacks at the same time. During the traverse of all sub-stacks, these somas would be repeatedly detected (green spheres in Fig. 5 A), which would produce many candidate somas. In these cases, we fused the repeatedly detected somas to obtain a final soma location. Similarly, because the average size of the somas was approximately 21 µm, adjacent somas with distances between their centroids of less than half of the soma size (10 µm) were fused. By traversing all somas and checking the distances between all somas, we obtained the final locations of all somas (red spheres in Fig. 5 B).

### Detection of Soma Surface

The original Rayburst Sampling method was initially proposed for the analysis of volumetrically complex shapes (such as spines) [25]–[27] in fluorescent images in which spines have greater grayscale intensities than the dark background. In Rayburst Sampling, the spine surface is detected by casting discrete rays from a certain point inside the spine. Principally, the ray would terminate when the intensity of the ray’s front point was lower than a specific designated threshold. Hence, once any ray was transmitted from the spine surface into the background, the ray was terminated by criteria based on a simple image intensity threshold. That is, this method is suitable for the detection of the surfaces of solid, blob-like objects. In the present work, some hollowness existed in several somas due to uneven staining. If we used the original Rayburst Sampling for our images, the criteria based on an intensity threshold would lead to the premature termination of the rays inside the soma, which would result in false detection of soma surfaces. Thus, the original Rayburst Sampling method did not satisfy our requirements.

**Figure 6:**
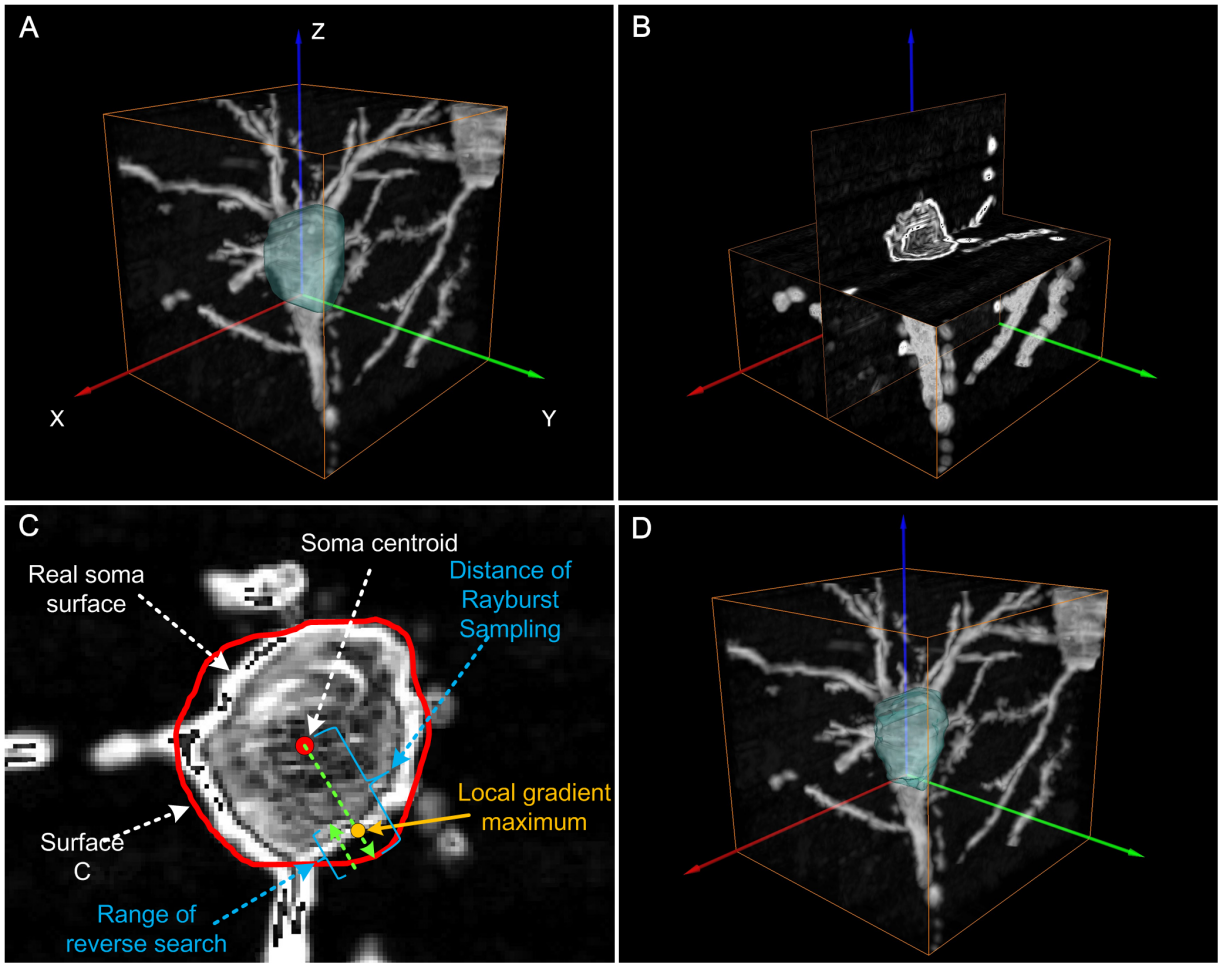
The algorithm for soma surface detection. (A) The surface (green) of the binary volume intersection (Fig. 4 E) obtained by the original Rayburst Sampling method was overlaid on the extracted image sub-stack. (B) The 3D gradient image shown in Amira in orthoslice view was computed from the image stack in (A). (C) Gradient-based Rayburst Sampling. A series of discrete rays (long green dashed line) was emitted from the soma centroid (red circle); the surface (red contour) of the volume intersection was obtained by the original Rayburst Sampling method. Then, a reverse search within a certain distance (short green dashed line) was conducted from each point on the surface along the reverse direction of ray casting to find a local gradient maximum (orange circle). Together, the positions of local gradient maxima made up the final detected soma surface. (D) The final detected soma surface (green) was overlaid on the extracted image sub-stack.

Given that a local gradient maximum based on image intensity often appeared near the soma surface (with the exception of soma locations connected with neurites), integrating the local gradient maximum as another criteria for ray termination was considered as a potential method to find the real soma surface. The remaining problem was how to identify the surface locations where the soma was connected with neurites. As mentioned before, a volume intersection (Fig. 4 E) larger than each soma had been generated by backprojecting three projection images into 3D image space. Notably, the volume intersection excludes almost all neurites outside itself (Fig. 6 A). Therefore, use of the volume intersection as a constraint for limiting the casting range of rays allowed successful detection of the real soma surface by finding the position of the local gradient maximum. Here, we refer to our improved Rayburst Sampling method as gradient-based Rayburst Sampling. Details of the algorithm are as follows.

Starting from the soma centroid, the original Rayburst Sampling method was used to find the surface (Fig. 6 A) of the binary volume intersection. The surface was overlaid on the original image stack.

A Sobel operator was used to calculate the 3D gradient image (Fig. 6 B) from the original image sub-stack.

Starting from every point on the surface of the volume intersection that was found in the first step, a local gradient maximum was determined (Fig. 6 C) along the reverse direction of ray casting within a certain distance. All local gradient maximum positions made up the finally detected soma surface (Fig. 6 D). Here, the distance for reverse search was empirically set to 3–4 µm.

The kernel used in the Sobel operator was a 2D matrix as follows 
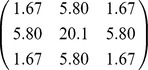



To calculate the gradient at a certain point in 3D image space, we first calculated the three gradient derivatives by convolving the 2D kernel with a local 3×3 neighborhood around the projection point in each of the three projection images. Then, the gradient magnitude of this point was found based on the three gradient derivatives, G_xy_, G_xz_, and G_yz_.

For all located somas in the large-scale image stack, the surfaces were obtained by repeating the procedure above on the sub-stack (60×60×60 µm^3^) around each soma. For each soma, the soma surface detected by gradient-based Rayburst Sampling was represented as a series of discrete points. We transformed the surface information into volume information that could be used for quantitative validation is explained in the following section.

### Quantitative Validation of the Proposed Method

To evaluate the performance of our proposed method, we compared the soma locations and surfaces detected with our proposed method with those generated by human segmentation.

(a) For validation of the accuracy of soma locations, the real centroids for all somas were first labeled manually by three experts. For each automatically located soma, we calculated the Euclidean distances between the current soma centroid and the centroids of the manually labeled somas. For somas with in which the Euclidean distance between the centroids generated by the two methods was less than a quarter of the average soma size (5 µm), the current soma location was considered to be correct. Finally, we used two parameters, Recall and Precision [31], [32], to quantitatively validate the accuracy of soma location: 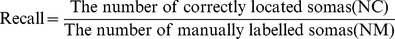


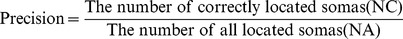



(b) To validate the accuracy of soma surface detection, the present work adopted the method of volume overlap measurement [33]. The volume overlap ratio for each soma was calculated using the following formula: 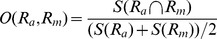



where *R_m_* is the region of soma volume segmented manually, and *R_a_* is the region of soma volume that was automatically detected. The operator calculates the common part of the two regions, and S(·) is the volume of the region.

Additionally, before we began manual segmentation of the soma volumes, we clarified the following issue: how to define the border between the soma and its connected neurites for an entire neuron, especially for those pyramidal neurons with apical dendrites that slowly change in diameter (such as the neuron in Fig. 6 A). To the best of our knowledge, no literature exists regarding this issue. In the present work, we assumed the smooth, blob-like structure that remained after all protruding neurites connected with soma were eroded as the soma region.

## Results

### The Results of Soma Location and Surface Detection

**Figure 7:**
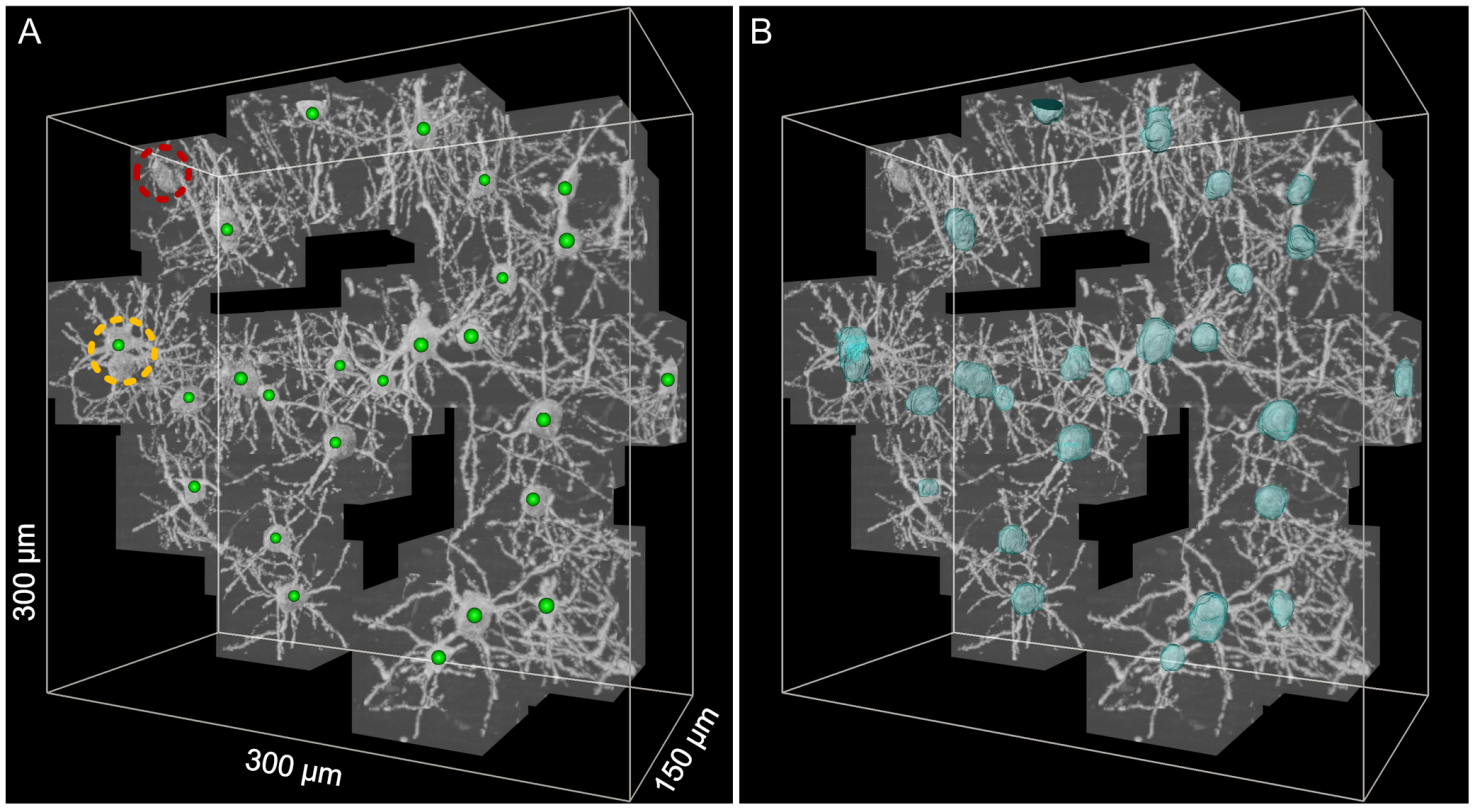
The results of soma location and surface detection for image stack 1. (A) The located soma centroids (green spheres) for image stack 1 were overlaid on the extracted image stack. The red circle indicates a missing soma; the yellow circle indicates the falsely detected soma (two somas were detected as only one soma). (B) The detected soma surface (green) for image stack 1 was overlaid on the original image stack. The automatically detected soma surfaces are shown in transparent blue and overlaid on the extracted image stacks.

In present work, we chose three image stacks with different sizes to test our proposed method for soma location and surface detection. The final soma locations and surfaces were overlaid on the original image stacks for visual display because comparatively dense neurites existed in each image stack, and this may be a disruption that would prevent us from seeing the results clearly. A local volume (60×60×60 µm^3^) around each real soma location was extracted from the original image stack to generate a new extracted image stack, which helped to show the soma location and surface more clearly. For a clear demonstration, we mainly show the results for the smallest image stack (Fig. 7). Additionally, Videos S1 and S2 demonstrate the surface detection results of the somas in image stack 1 (Video S1) and 2 (Video S2); Fig. S1 shows the soma localization result for image stack 3.

### Quantitative Validation of Soma Locations

For the manually labeled soma positions and volumes, we did not take into account somas that were broken at the borders of image stacks. The sizes and soma localization results for the three image stacks are shown in Table 1.

**Table 1:** Validation of proposed method for soma location using three image stacks.

Image Stack	Size ( µm^3^)	Real soma number	Correct Detection	False Detection	Precision	Recall
1	300×300×150	27	24	1	96.0%	88.9%
2	525×525×180	72	72	0	100%	100%
3	1000×1000×180	211	195	11	94.6%	92.4%
Total		310	291	12	96.0%	93.9%

The main drawback of our proposed method for soma localization is that two close somas (as indicated by the yellow circle in Fig. 7) can appear as a cluster in the projection images, and these clusters were not well distinguished by our multi-scale morphological closing.

### Quantitative Validation of Soma Surface Detection

**Figure 8:**
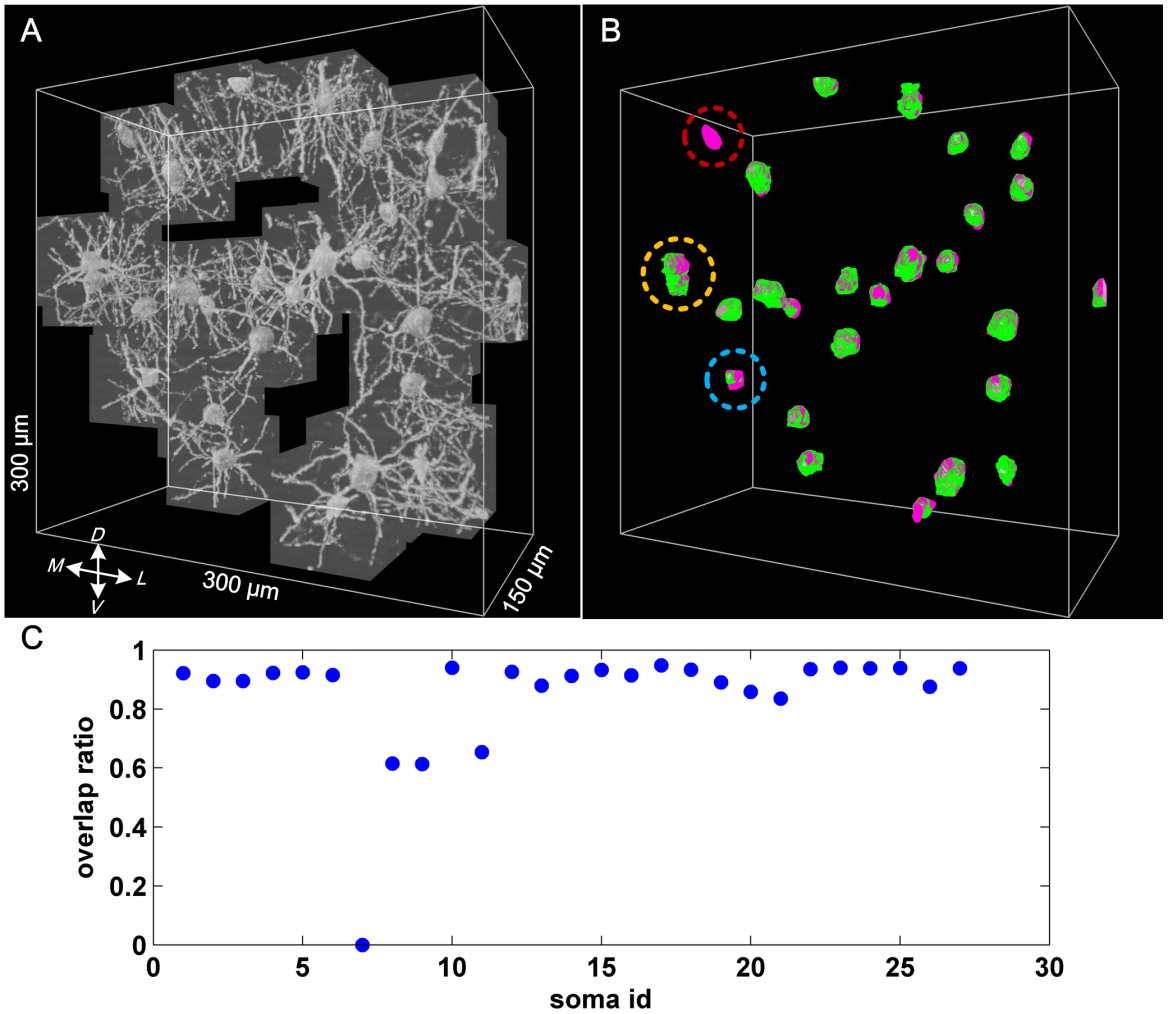
Validation of soma surface detection in image stack 1. (A) The image stack 1 including all extracted image sub-stacks. (B) The automatically detected soma volumes (green) were overlaid on the manually segmented soma volumes (magenta). The soma in the red circle was missed; thus, the volume overlap ratio was zero. The two closely spaced somas in the yellow circle were falsely detected as a single soma; thus, the automatically detected soma volume included both of these two somas. The sickle-like soma in the blue circle was too irregular to be detected accurately. (C) The volume overlap ratio for all 27 total somas.

Because manual segmentation of soma volumes is time consuming, we chose only two relatively small image stacks for manual segmentation. Notably, only those correctly located somas could be used to calculate the volume overlap ratio. If any soma was missed, the related volume overlap ratio was set to zero. The final validation results are shown in Table 2 and Fig. 8.

**Table 2:** Validation of the proposed method for soma surface detection using two image stacks.

Image Stack	Size ( µm^3^)	Real soma number	Correct Detection	Overlap ratio >80%	Overlap ratio >84%
1	300×300×150	27	24	23 (96%)	23 (96.0%)
2	525×525×180	72	72	68 (94.4%)	63 (87.5%)
Total		99	96	91 (94.8%)	86 (89.6%)

For the 27 total somas in image stack 1, the volume overlap ratio for the soma labeled 7 (soma id 7) was zero; this finding corresponded to the missed soma indicated by the red circle in Fig. 7 B. For the two somas labeled 8 and 9, the volume overlap ratios were about 0.6, which corresponds to the two somas indicated by the yellow circle in Fig. 7B. These two somas were too close to be distinguished by our proposed method. For the soma labeled 11, the volume overlap ratio was a little larger than 0.6, which corresponds to the soma indicted by the blue circle in the Fig. 7 B. This sickle-like soma was too irregular to be detected accurately.

Similarly, to show our results more clearly, the extracted image stacks mentioned above were overlaid with our soma surface detection results. Additionally, Fig. S2 shows the result of soma localization and the validation result of surface detection for image stack 2.

### Computational Efficacy, Hardware, and Software

We implemented the proposed method using C++ for the Windows platform. The volume and surface rendering for some intermediate results were implemented using commercial Amira software (Visage Imaging GmbH). The source code and test dataset will be open to the public in a near future. The tests were performed using a Windows workstation with an Intel(R) Dual Core(TM) Xeon 3.47 GHz CPU with 48 GB RAM. Our soma detection program took approximately 2 minutes to process a 3D image stack of 900×900×450 and 5 minutes for an image stack of 1500×1500×540.

## Discussion

Overall, the bottleneck in the computational efficiency of the proposed method lies in the size of the original image stack and the number of somas in the image stack. So, the complexity of our algorithms has an approximately linear relationship with the product of image volume size and soma numbers (width * height * depth * soma_numbers). This can be acceptable by general application. It may not be a good strategy to traverse each image sub-stack in sequence when only few somas exist in a comparatively large image stack. In such cases, integrating specific human interactions into our proposed method would be a better choice. Indeed, the deblocking strategy used in our method make it possible to parallelize our method for reducing computing times. But it will be our next work. Temporarily, some parallel algorithm steps during the soma detection in each sub-block have been implemented using OpenMP technique in our present algorithm version.

For the soma localization, the multi-scale morphological closing used in present work may fail in the cases in which multiple somas are close to each other. Fortunately, the frequency of these cases is low in Golgi staining images. When such cases appear, they can be overcome by moderate human interaction.

As for the soma surface detection, surface detection for two types of somas needs to be refined in future work. On the one hand, the proposed gradient-based Rayburst Sampling is ideal for those somas with sphere-like surface morphologies. For somas with irregular surface morphologies, such as slender or sickle-like somas, even the morphological close based on the smallest template size could not obtain a volume intersection containing the entire soma in the present work. In general, comparatively less hollowness occurs in these somas according to our large amount of statistics. In these cases, we could use another variant of the present gradient-based Rayburst Sampling that does not include volume intersection as a constraint. That is, the real soma surface may be found by searching for the first local gradient maximum larger than a certain gradient threshold along the ray casting that starts from the soma centroid. On the other hand, as mentioned before, Golgi staining may result in hollowness in somas. However, in some cases, holes appear on the soma surface. A satisfactory local gradient maximum may not be found near these holes.

In order to use our algorithm correctly, some important parameters need to be determined in advance according to the feature of neuron images. In this paper, a statistical experiment was carried out to determine the minimum, maximum, and average soma size in a specific neuron images. Then this information is used to determine the overlap size of each sub-stack, the range of multi-scale morphological closing, and the threshold for soma fusion. Generally, these parameters mainly depend on the resolution of neuron images. So, we have constructed a relationship between the image resolution and these parameters in this algorithm. Any user who wants to use our algorithm can start using it by simply setting the resolution information of their specific neuron images.

The proposed method is suitable for neural image stacks with random and sparse soma distributions. Given the widespread use of fluorescence proteins in neuroscience, more and more sparse neuron labeling has been consistently achieved [5]. Furthermore, there is frequently no hollowness in the somas of fluorescence neuron images. Although our method is proposed based on Golgi stained neuron images, our method can be readily adapted for soma detection in general fluorescence neuron images.

### Conclusions

The main outcome of the present work is a new method for the location and surface detection of multiple somas with hollowness and variable sizes in large-scale neural image stacks that contains a relatively sparse soma distribution. First, detection of multiple somas in large-scale neural image stacks was achieved by image deblocking that included overlaps between adjacent image sub-stacks. Second, somas with hollowness and variable sizes were automatically located by integrating multi-scale morphological closing and adaptive thresholds into the 2.5 D morphological method. Third, assisted by the constraint of volume intersections generated by 2D image backprojection, accurate surface detection of somas with hollowness and connected neurites was successfully achieved by integrating a new gradient-based criteria into the classical Rayburst Sampling method.

Automated and accurate soma localization can not only be useful for the study of soma distributions in large-scale neural networks but also could supply ideal seed points for seed-based neurite tracing algorithms [34]. The soma surface is one of the most important factors for discriminating different neuron types. The proposed method for soma surface detection offers a good foundation for the systematic study of neuron types in different brain regions based on large-scale neural image datasets.

## Supporting Information

Figure S1 **Soma localization result for image stack 3**. The automatically located soma centroids (green spheres) and manually labeled soma centroids (magenta spheres) are overlaid on the extracted image stack.
(JPG)

Figure S2 **Results of soma localization and surface detection for image stack 2**. (A) The automatically located soma centroids (green spheres) are overlaid on the extracted image stack. (B) The automatically detected soma volumes (green) are overlaid on the manually segmented soma volumes (magenta). (C) The volume overlap ratios for all 72 somas.
(JPG)

Video S1 **Surface detection result of somas in image stack 1**. The automatically detected surface (green) was overlaid on the extracted image stack.
(MOV)

Video S2 **Surface detection result of somas in image stack 2**. The automatically detected surface (green) was overlaid on the extracted image stack.
(MOV)
