# Supplementary figures and images for: Automated and Accurate Detection of Soma Location and Surface Morphology in Large-Scale 3D Neuron Images
Source: PLoS One. 2013 Apr 24;8(4):e62579. doi: 10.1371/journal.pone.0062579 (PMC3634810; doi:10.1371/journal.pone.0062579)

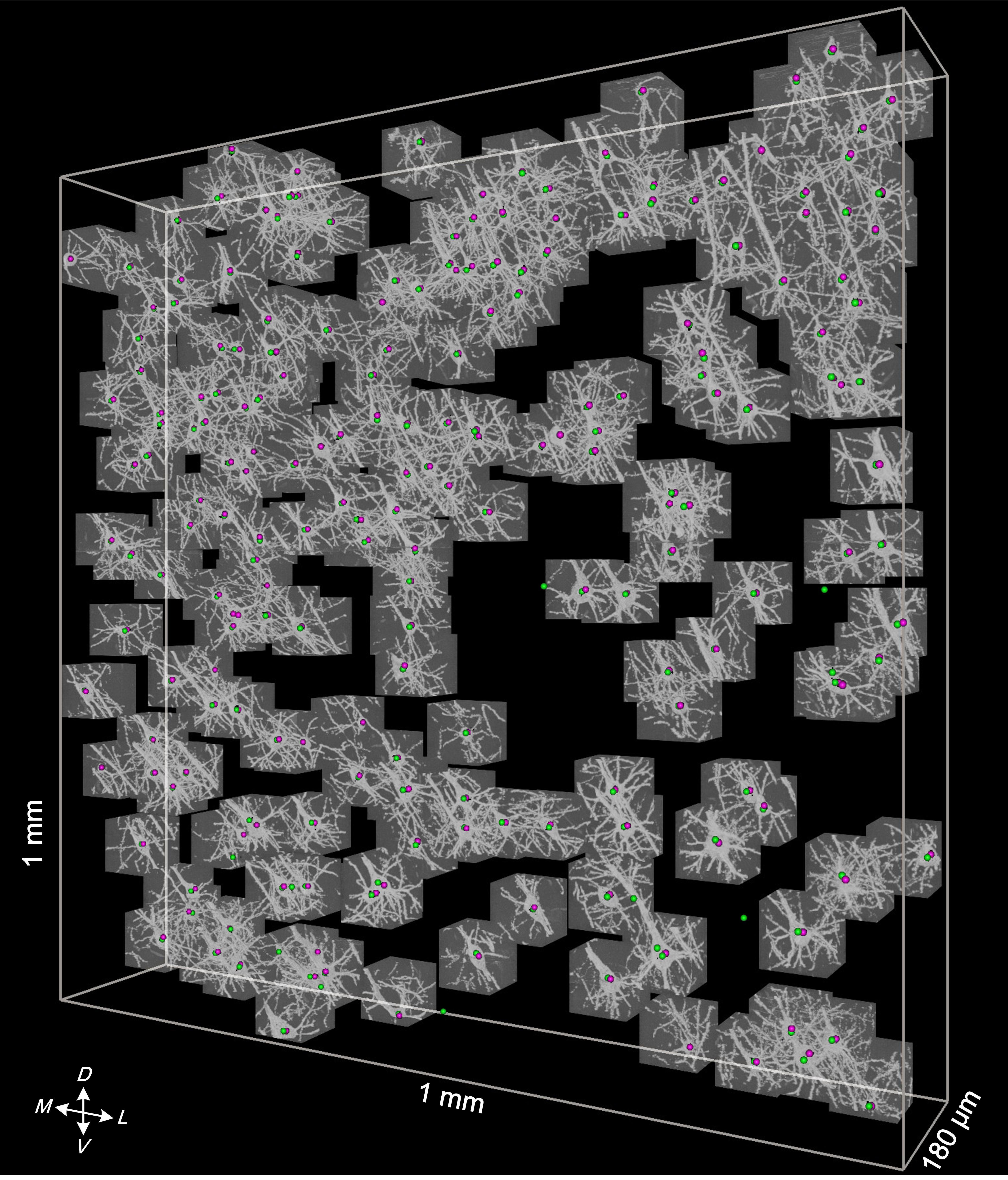

Supplement: Figure S1 — Soma localization result for image stack 3. The automatically located soma centroids (green spheres) and manually labeled soma centroids (magenta spheres) are overlaid on the extracted image stack. (JPG) [file pone.0062579.s001.jpg]

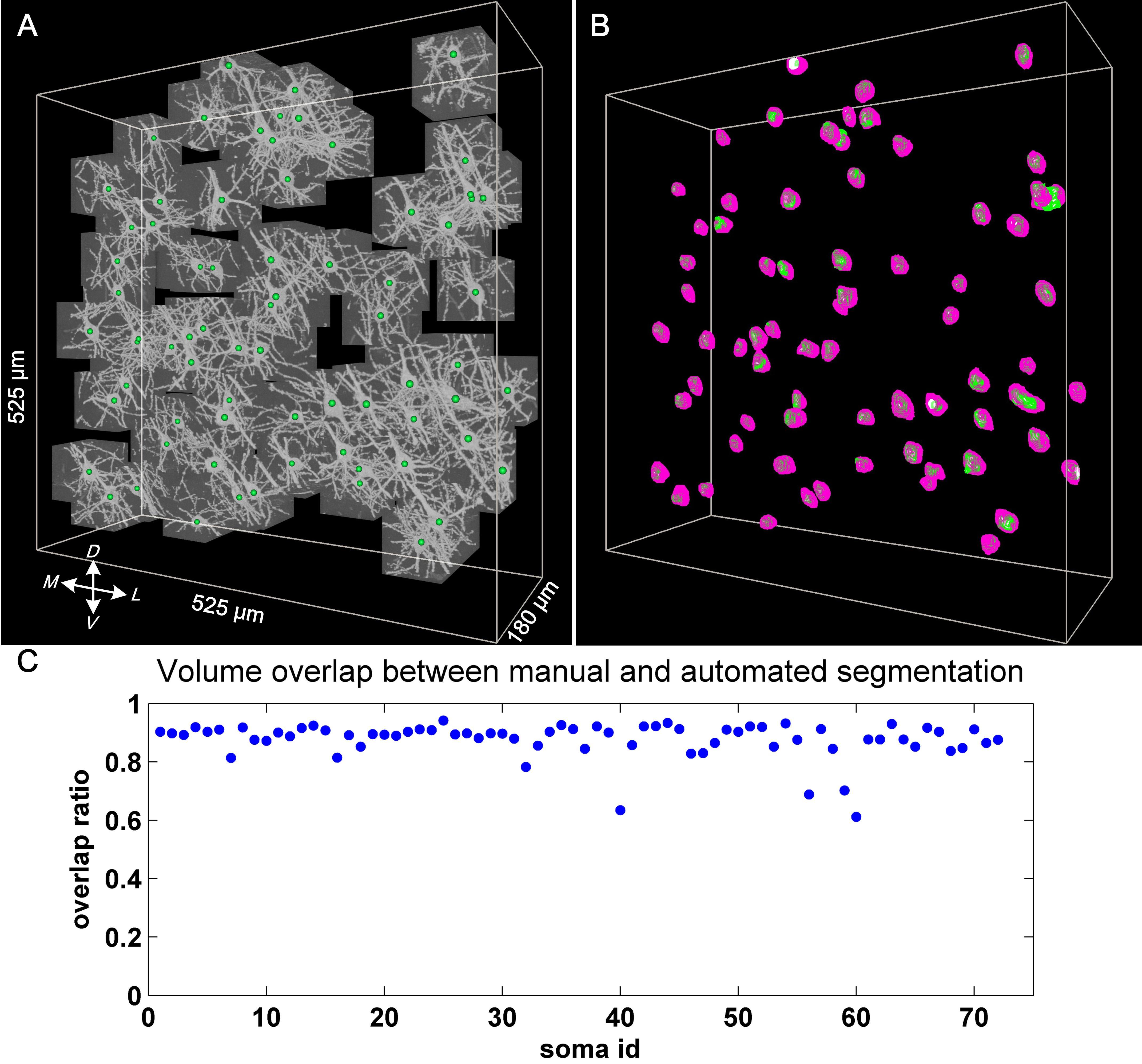

Supplement: Figure S2 — Results of soma localization and surface detection for image stack 2. (A) The automatically located soma centroids (green spheres) are overlaid on the extracted image stack. (B) The automatically detected soma volumes (green) are overlaid on the manually segmented soma volumes (magenta). (C) The volume overlap ratios for all 72 somas. (JPG) [file pone.0062579.s002.jpg]
